# Molecular, biochemical, and sensorial characterization of cocoa (*Theobroma cacao* L.) beans: A methodological pathway for the identification of new regional materials with outstanding profiles

**DOI:** 10.1016/j.heliyon.2024.e24544

**Published:** 2024-01-26

**Authors:** Andrea Zapata-Alvarez, Carolina Bedoya-Vergara, Luis D. Porras-Barrientos, Jessica M. Rojas-Mora, Héctor A. Rodríguez-Cabal, Maritza A. Gil-Garzon, Olga L. Martinez-Alvarez, Carlos M. Ocampo-Arango, Maurem P. Ardila-Castañeda, Zulma I. Monsalve-F

**Affiliations:** a University of Antioquia, Faculty of Exact and Natural Sciences, Institute of Biology, Agrobiotechnology Research Group, Calle 67 N°. 53 - 108, A.A 1226, Medellín, Colombia; b La Sallista University Corporation, Caldas, Antioquia, Colombia, Food Engineering Research Group, GRIAL, Carrera 51 N°.118 sur 57, Caldas, Antioquia, Colombia; c University of Antioquia, Faculty of Pharmaceutical and Food Sciences, Sensory Science Research Group, Calle 67 N°. 53 - 108, A.A 1226, Medellín, Colombia; d Metropolitan Technological Institute, Faculty of Exact and Applied Sciences, Medellín, Colombia

**Keywords:** *Theobroma cacao* L, Colombia, Fine flavor, Genetic diversity, Antioxidant capacity

## Abstract

Cocoa is an economically important product in Colombia. On-farm germplasm evaluations enable the selection of superior genotypes for propagation and distribution across the country. This study examined 12 cocoa samples from Antioquia along with five reference materials, employing 96 single nucleotide polymorphism (SNP) markers. Furthermore, these genetic findings were correlated with physical, chemical, and sensory attributes. Primary coordinate analysis revealed that the majority of samples were hybrids derived from five original germplasm pools, including Criollo, Amelonado, and three Upper Amazon Forastero cocoas. The integral profile of the 12 selected materials was classified into Modern Criollo (Rodriguez-Medina et al., 2019) [3], Forasteros (Rodriguez-Medina et al., 2019) [3], and Trinitarios (Borja Fajardo et al., 2022) [6]. Three key factors were identified to best account for the sample classification: type of variety, functional properties, and quality.

## 1 Introduction

Cocoa (*Theobroma cacao* L.) is a small-sized plant [1] that is native to the Amazon basin and whose cultivation has been important in several Latin American countries since colonial times. Cocoa is produced for export purposes, particularly to European countries that use it as a raw material for the production of chocolates. In Latin America, cocoa cultivation in Brazil is the most important as it represents 40 % of the total regional production in terms of planted hectares. The cocoa production of other Latin American countries in order of planted hectares are as follows: Ecuador (24 %), Colombia (9 %), the Dominican Republic (9 %), Peru (6 %), and Venezuela (4 %). In 2016, income from the export of cocoa reached 1.65 billion US dollars [2].

The increased consumption of chocolate worldwide provides an opportunity to strengthen the entire value chain of cocoa producing countries, for which cocoa has become a catalyst for economic growth and is considered an alternative to illicit crops or crops associated with climate change [3].

Cultivated cacao has traditionally been subdivided into three main groups, Criollo, Forastero, and Trinitario [4,5]. Many Criollo hybrids (known as Trinitario) are recognized as “Finos de Aroma” according to the International Cocoa Organization (ICCO). “Fine Aroma” cocoa accounts for approximately 8 % of world production [5,6] and describes a material with an exquisite aroma and flavor of fruity, floral, nutty, and malty [7] making it the preferred raw material for making premium chocolate. However, a substantial portion of these hybrids are, in turn, the offspring of such crosses. A smaller proportion is established with genetic materials sourced from Forastero clones [8], thereby showcasing a noteworthy diversity in the cocoa ecotypes cultivated in Colombia. Despite the emphasis on promoting cocoa cultivation with desirable sensory attributes, it's worth noting that the potential of Colombian cocoa has traditionally been assessed based on its agronomic characteristics. This approach has certainly facilitated effective crop management; however, it doesn't encompass other selection considerations that could contribute to generating added value in the end products [9].

Currently, between 15 % and 44 % of cacao accessions or materials are estimated to be mislabeled [10]. Accordingly, simpler identification techniques based on genetic markers have been used for genetic diversity studies, detecting mislabeled products, and establishing the genetic relationships within and between populations or individuals. SNP assays can be performed without separating DNA according to size and can therefore be automated in a high-throughput assay format. In addition, the di-allelic nature of SNPs confers a much lower error rate than the use of other molecular markers.

Based on data from Fedecacao (Colombia's National Cocoa Fund) and ICCO, approximately 95 % of Colombian cocoa beans earmarked for export, which equates to 55,000 tons, have been certified as Fine Aroma cocoa. In 2017, researchers from Corpoica and CIAT (International Center for Tropical Agriculture) successfully determined the genetic variability and population structure among cocoa accessions from the Banco de Germoplasma de Corpoica (Colombian Corporation of Agricultural) using diversity results obtained through Fluidigm technology [11]. This technology relies on a microwell-based platform employing a limited set of SNP markers [12]. The benefits of this approach encompass reduced execution cost, enhanced performance, and simplified reaction configurations [13]. The identification of characteristics conducive to the creation of genetic classifications of cocoa may have utility in improving the cultivation of cocoa and other key crops in developing countries such as Colombia.

The objective of this study was to assess the genetic diversity, chemical composition, and sensory attributes of cocoa materials cultivated in three distinct sub-regions of Antioquia, namely Bajo Cauca, Magdalena Medio, and Urabá, Colombia. This investigation aimed to establish a foundational framework for the preservation and utilization of cocoa germplasm originating from this region. To achieve this, a comprehensive methodology was employed to identify cocoa materials demonstrating exceptional quality and possessing the potential for registration and subsequent propagation.

## 2 Materials and methods

### 2.1 Sample origin

**Fig. 1:**
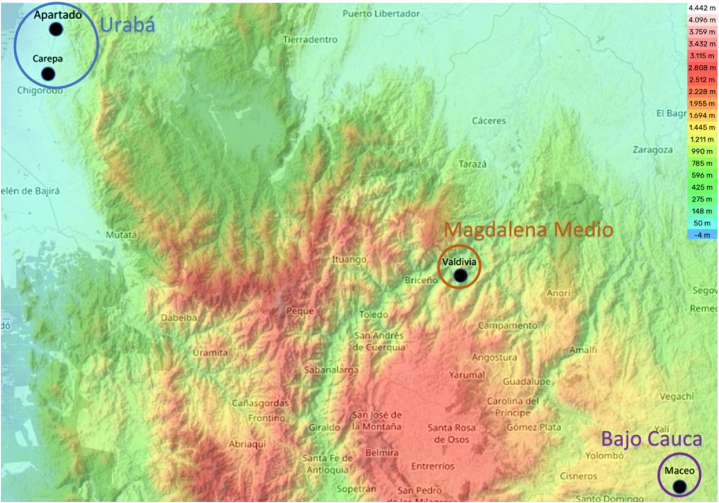
Map of sample collection sites. Sampling sites were selected to cover different planting altitudes. The characteristics of each region are elaborated upon in Supplementary Data 1 (S1). We have enhanced the Antioquia map using free topographic data from topographic-map.com, which includes information on elevation, relief, and topography.

A total of 12 materials were meticulously chosen from plants identified by Fedecacao as part of the project CPT-1502. These particular materials were singled out as distinctive and exceptional due to their noteworthy agronomic attributes and flavor profiles of the beans, both pre- and post-harvest. Notably, these cocoa fruits lacked a homologous counterpart in the planted region. As a result of this unique characteristic, their potential to be included in future studies for registration and subsequent propagation was recognized. These determinations align with the stipulated regulations set forth by authorized organizations responsible for such recognition [14]. All selected materials were sourced from farms situated in the key cocoa-producing municipalities: Bajo Cauca (one municipality), Urabá (four municipalities), and Magdalena Medio (seven municipalities) (Fig. 1, S1)..

### 2.2 Molecular characterization

#### 2.2.1 Extraction and DNA preparation

The young leaves of the 12 selected materials were cut, placed in Ziploc bags, and stored in dry ice until they reached the laboratory and were stored at −80 °C. DNA was extracted from the leaves of cocoa trees at the Plant Molecular Biology Laboratory (Universidad de Antioquia, Colombia) using a modified CTAB protocol [15]. DNA concentration was quantified using a NanoDrop 1000 spectrophotometer (NanoDrop Technologies). DNA samples with absorbance ratios greater than 1.8 at 260/280 nm (nm) and greater than 1.2 at 260/230 nm were used for SNP marker analysis at CIAT (International Center for Agricultural Tropical, Palmira-Cali, Colombia).

DNA quality was analyzed following the practical guide for SNP genotyping using the EP1 and SNP type assay system from Fluidigm version F_03 [16] that comprises agarose gel electrophoresis (0.8 % agarose, 0.5X TBE and stained with SYBR Safe) and quantification by spectrophotometry (Synergy-H1 m). DNA purification was performed using RNAse A to remove RNA present in samples. For genotyping, the samples with the highest quality were chosen and adjusted to a final concentration of 60 ng/μL.

#### 2.2.2 SNP markers and genotyping

A set of 96 SNP markers was used, of which 48 were developed by researchers from the United States Department of Agriculture (USDA) and Fluidigm Corp. (San Francisco, California, USA) [[17], [18], [19]]. The remaining 48 SNP markers were requested from the Tropgene database of the CIRAD Research Center (Ruiz et al., 2004). Genotyping was performed on a Fluidigm EP1 platform according to an allele-specific detection methodology, SNPtype, developed by Fluidigm and adapted at CIAT [11]. Genomic regions containing SNPs were amplified by PCR (Polymerase Chain Reaction) to establish PCR templates. PCR templates and primers for each SNP marker were transferred to a solid support called an Integrated Fluidics System (IFC). Once loaded or injected into the central part of the IFC, allele-specific PCR was performed. Results were determined by the capture of fluorescent signals as images and analyzed with Fluidigm SNP software for genotyping analysis version 4.1.3 [20]. Four internal controls from known genetic groups were included during data collection but were not considered for further analysis.

#### 2.2.3 Data analysis

The Fluidigm SNP Genotyping Analysis Software program version 4.1.3 presents a graph for each SNP where all individuals are grouped as homozygous or heterozygous depending on fluoresecent intensity. The program also provides a matrix with the genetic profiles of each evaluated material. To assess the relevance of the SNP markers used, descriptive statistical analyses of major allele frequency, genetic diversity, heterozygosity (H), and polymorphic information content (PIC) were performed using PowerMarker V3.25 software [33]. To observe the distribution of the samples, paired genetic distances were calculated in PowerMarker by comparing the genetic profiles and transforming the differences found into distance values [34]. These distances were obtained following different standards according to the nature of the data according to the principle of the Rogers standard (1972) [35], which does not assume a priori evolutionary forces and is considered adequate for the evaluation of breeding collections [36]. This standard distributes samples in a range from 0 to 1, where 0 indicates no genetic differences and 1 indicates total differentiation between individuals. The resulting numerical matrix was grouped into a dendrogram or distance tree using the UPGMA algorithm, where the lengths of each branch indicate the genetic distance between individuals. The resulting trees were edited in FigTree V1.4.2 [37]. Finally, a principal coordinates analysis was performed using GenALEx V6.502 [38] to transform the obtained distance data into coordinate values which allowed the samples to be plotted in a Cartesian plane according to their genetic relationship.

#### 2.2.4 Principal component analysis

Principal component analysis (PCA) was used to analyze the data set based on similarities and differences by transforming the original variables into a new set of uncorrelated variables without losing information based on linear combinations that maximize variability [39]. The PCA started with the analysis of a matrix (17 × 8) consisting of 17 samples (rows) composed of five cocoa references materials (CCN51, ICS1, IMC67, FLE2, and FEC2) and 12 new cocoa materials from Antioquia, Colombia (E8061, E1162, E1182, E1192, E1242, E1312, E2013, E2053, E2073, E4023, E4063, and E6063). The columns [8] contained values of the evaluated variables (ethereal extract, total polyphenolic content, total anthocyanin content, ORAC, theobromine, caffeine, ratio of theobromine to caffeine, and sensory quality). All analyses were performed in triplicate. A Bartlett sphericity test was performed to exclude the presence of significant correlations between variables in the correlation matrix.

#### 2.2.5 Principal factor analysis

The previous PCA and the analysis of common factors for each studied material was performed using the Kaiser–Varimax method transformation criterion. Factors that resulted in axes with few large loads and loads of as close to zero as possible on other axes were identified using iterative maximization of the quadratic function of loads. Multivariate analysis was performed using the statistical software, R (Version 0.98.1103 GNU Affero General Public License; https://cran.r-project.org).

### 2.3 Biochemical characterization

#### 2.3.1 Sample preparation

The cocoa pods were previously washed and sanitized before opening and removing all the seeds. The unfermented cocoa beans without mucilage were frozen at −20 °C and then ground (Hamilton Beach, model no. 80370, USA) to a size between 600 and 420 μm (40 mesh). The resulting powders were lyophilized in a Christ alpha 1–2 LO plus equipment, the main drying was at −50 °C and 0.04 mbar for 14 h and the final drying −76 °C, at 0.01 mbar for 14 h, the final moisture content was between 1 % and 3 %. Each of the cocoa powder samples at minimum moisture were packed in screw cap centrifuge tubes, which were placed inside a plastic box containing silica gel to prevent moisture absorption during storage at −20 °C, until they were used in all the chemical analyses described in this section 2.3.

Samples were defatted for all chemical analyses except the determination of total anthocyanin content. Defatting was performed using the method reported by Carrillo et al. [21] (with slight modifications. 100 mg of sample was weighed in a plastic reaction tube with 1.5 mL of hexane and was taken to an ultrasonic for 15 min at 25 Hz, 99 % frequency and 25 °C, 99 % frequency and 25 °C. It was then centrifuged at 14000 rpm, 18 min and 15 °C. The supernatant was removed, and the procedure was repeated four times combining the supernatants. Finally, to obtain total EE, the solvent was evaporated at room temperature in laboratory fume hood [21].

#### 2.3.2 Ethereal extracts

The quantification of ethereal extracts at low temperature was performed according to the method described by Carrillo et al. [21] with slight modifications. In the present study, 100 mg of each sample was placed in a plastic reaction tube with 1.7 mL of hexane and incubated in an ultrasonic bath for 15 min at 25 °C with application of a 25 Hz frequency at 99 % power. Samples were then centrifuged at 14000 rpm for 15 min at 15 °C. The supernatant was removed and the procedure was repeated four times. The resulting supernatants were combined to obtain the total ethereal extract. The solvent was then evaporated at room temperature in a laboratory fume cabinet [21].

#### 2.3.3 Extraction of antioxidant compounds and alkaloids

For the extraction of antioxidant compounds and caffeine, 100 mg of defatted cocoa was mixed with 1.7 mL of acetone/water MilliQ/acetic acid solution (70/29.5/0.5, v/v/v) for 1 min using a Vortex mixer and then incubated in an ultrasonic bath for 15 min at 25 °C with the application of a 25 Hz frequency at 99 % power. Samples were then centrifuged at 14000 rpm for 10 min at 10 °C. The supernatant was collected and the procedure was repeated four times. Both extracts were then combined and used for the determination of total contain of polyphenols, antioxidant capacity and caffeine [22].

#### 2.3.4 Total phenolic content

Total phenolic content (TPC) was determined using Folin-Ciocalteu reagent [23]. Briefly, 30 μL of gallic acid (10, 20, 40, 60, 80, or 100 μg/mL) and 30 μL of the sample diluted with distilled water at a ratio of 1:60 were added to a 96-well plate polystyrene microplate (Costar, United States). After 6 min, 30 μL NaCO2 (10 %w/v) was added, followed by 15 μL of Folin-Ciocalteu reagent except for the blank (water). Mixtures were then incubated at 25 °C for 60 min and absorbance was measured at 760 nm using a Synergy HT plate reader (Biotek Instruments Inc, USA). TPC was expressed as the equivalent of 1 mg of gallic acid per gram of dried sample material (mg GAE/g dried sample). All measurements were performed in triplicate.

#### 2.3.5 Determination of antioxidant capacity using the oxygen radical absorbance capacity assay

The Oxygen Radical Absorbance Capacity assay was performed based on previous reports by Londoño [24] with some modifications. The excitation intensity (I) was read at 493 nm and slit of excitation 5 and the emission intensity (I) was read at 515 nm and slit of emission 13, with attenuator of 1 % and without attenuator plate [24].

Antioxidant protection was measured from the fluorescence area under the curve calculated according to equation (1):

(1) $$\left(0,5+\left(\sum_{i=1}^{i=60}\frac{f_{i}}{f_{1}}\right)\right)x\;\text{CT}$$

i: number of cycles, f: fluorescence units, CT: time of each cycle in minutes (CT = 2)

The protective effect of the antioxidant was calculated from the differences in areas under the decay curve of fluorescein between the blank and the sample (AH). This value was then compared to the Trolox curve and expressed in micromoles of Trolox per gram of sample (mmol Tx/g sample), according to equation (2):

(2) $$\text{ORAC}=\frac{{\text{AUC}}_{\text{AH}}}{{\text{AUC}}_{\text{Trolox}}}x\frac{\left[\text{Trolox}\right]}{\left[\text{AH}\right]}$$

#### 2.3.6 Total anthocyanin content

The total content of anthocyanins was according to a previously described method [25,26] with slight modifications. A total of 0.1 g of ground and sieved cotyledon was subjected to extraction with 10 mL of methanol and HCl at a ratio of 97:3. The mixture was left at 8 °C for 17 h. Then, 200 μL of the supernatant was removed with a micropipette and added to a 96-well quartz microplate (Costar, United States) and the absorbance ratio at 460 nm and 530 nm was measured using a Synergy HT spectrofluorometer (Biotek Instruments Inc, United States). Readings were performed in triplicate.

#### 2.3.7 Determination of alkaloid content

##### 2.3.7.1 Extraction

Caffeine was extracted following the method described in Section 2.3.3 and theobromine was extracted using a method based on some parameters reported by Ref. [27] with previously described modifications [28]. Extraction from 50 mg of previously defatted cocoa was performed with the addition of 1.5 mL of a solution of NaOH in ultrapure water at a pH of 10 at 90 °C using an ultrasound-assisted method. The solution was vortexed for 15 min at 3000 rpm and the compounds were extracted by ultrasound at 40 min, 25Hz and 50 °C, conditions optimized after evaluating temperature (20, 30 and 50 °C) and frequency (25, 40 and 59 Hz). Solid phase separation was performed by centrifugation at 14000 rpm for 15 min and the supernatant was removed. The procedure was repeated on the remaining solid, 3 more times. The supernatant collected from the four extractions was volumetrically gauged to a volume of 8 mL and filtered through a 0.22 μm Nylon membrane. Finally, 400 μL of the final extract was dissolved in a vial with the same amount of solvent, which in this case consisted of the mobile phase of the chromatographic method (0.1 % formic acid solution and acetonitrile in a 95:5 ratio, respectively).

##### 2.3.7.2 Quantification

The quantification of alkaloids was performed according to a previously described method [22] with optimized modifications [28]. For the identification of standards and samples, MS/MS detection was performed in positive mode. Transitions were evaluated by selected reaction monitoring (SRM) (Table 1).

**Table 1:** Optimal selected reaction monitoring (SRM) conditions for the identification and quantification of alkaloids using UHPLC-MS/MS.

Compounds	SRM 1 (quantification)	Cone voltage	Collision energy	SRM 2 (confirmation)	SRM 3 (confirmation)	Cone voltage	Collision energy
Caffeine	194.985 > 137.97	44	18	194.985 > 41.879	194.985 > 109,929	44	22
Theobromine	180.905 > 162.922	36	20 15	180.905 > 66.917	180.905 > 107,573	36 45	20 10

The parameters of the ionization source for triple quadrupole detection were a capillary voltage of 3 kV, source temperature of 150 °C, solvation temperature of 400 °C, flow velocity of solvation gas (nitrogen) of 650 L/h), and flow velocity of ionization gas (argon) of 80 L/h). The stock solution of caffeine was dissolved in acetonitrile at a concentration of 1000 mg/L. For the preparation of a calibration curve in the range of 0.02–50 mg/L, 600 μL of the mobile phase was added to correct for the matrix effect and calibrated with the mobile phase (0.1 % formic acid solution and acetonitrile at a 95:5 ratio) with application of the coefficient of determination for caffeine (0.999282). Theobromine required the preparation of a solution with a maximum concentration of 500 mg/L in Type I water at 90 °C, given its solubility, with stirring for 1 h at 90 °C in a water bath and sonication at 25 Hz for 5 s at 30 min. The working range of the standard 2.5 calibration curve was 100 mg/L (R2 0.999457). SRM 2384 baking chocolate (NIST, USA) was used as a chocolate reference material for standardization of the ether extraction of theobromine, caffeine, and theophylline.

### 2.4 Sensory analysis

Sensory panel was performed using a panel comprising three judges from the Sensory Science Research Group of the Universidad Antioquia with expertise in the olfactory and taste sensory analysis of cocoa fruits. Judges were trained under GTC 280: 2017: ISO 8586: 2014 Guidelines for the selection, training and monitoring of selected sensory evaluators and experts [29], through the use of olfactory references for cocoa that included compounds such as Isoamyl Alcohol, Benzaldehyde, Isovalerian Acid, Caproic Acid; in order to identify and memorize quality descriptors and cocoa defects. Cocoa quality and defect descriptors were identified using type clones from the different subregions of Antioquia and through the use of olfactory references under NTC 4503:2011: ISO 5496:2006 (Initiation and training of evaluators in the detection and recognition of odors) and NTC 3915:2012:ISO ISO3972:2011(Method to investigate the sensitivity of taste) [30,31].

The analysis of the study materials began with the opening of each of the twelve clones evaluated to subsequently extract the fresh grain. Approximately 5 g of sample were weighed, which were served in coded containers with three random digits and delivered individually to the judges who analyzed the appearance, smell and flavor descriptors and the general quality, based on quantitative descriptive analysis according to: 1994 NTC 3932:1996: ISO 11035 (Identification and selection of relevant descriptors that give the maximum information about the sensory attributes, to establish a sensory profile) [32].

The trained panel provided a quantitative descriptive analysis according to NTC 3932:1996: ISO 11035:1994 (Identification and selection of relevant descriptors that give the maximum information on sensory attributes, to establish a sensory profile) of the sensory attributes of odor (floral, sweet, acid, fruity, nutty, or woody) and taste (floral, sweet, acid, bitter, green, fruity, woody, nutty, astringent sensation, spiciness sensation, and general quality) [32]. Based on the intensity of the attributes, the judges established the general quality that was used as a correlation parameter, where the presence of a high intensity in fruit, floral and sweet descriptors indicated a high quality and the bitter and sensations of astringency and spiciness indicated a high quality. decrease in sensory quality. All descriptors were rated on a scale of 0–10, where 0 = not noticeable, 0.5–2.0 = beginning to be noticeable, 2.5–5 = moderate, 5.5–6.5 = medium high, 7 0.0–8.0 = strong/high and 8.5–10.0 = very strong/very high). The general quality of each fruit was evaluated by qualifying 16 descriptors between smell [6] and taste [10] on a scale of 0–10 where 0 = very low, 0.5–2.0 = low, 2.5–5 = medium/low, 5.5–6.5 = average, 7.0–8.0 = high, and 8.5–10.0 = excellent). Cocoas with a general quality of 7.0 or more were considered those with the greatest balance and sensory harmony. The trained panel also evaluated the absence of defects (mold, green, earthy, chemical). The evaluation was carried out under controlled conditions of temperature of 24 ± 0.2 °C and relative humidity between 53 and 60 %.

## 3 Results and discussion

### 3.1 Molecular characterization

#### 3.1.1 SNP genotyping

The final 93 markers were shown to be polymorphic with an average PIC of 0.299. The average heterozygosity value (H) of the samples evaluated in the present study was 0.388, which is very close to the reported heterozygosity (H = 0.39) for cocoa samples in Colombia and Ecuador [4]. These results indicate that the SNPs used in the present study were informative in the evaluation of the genetic diversity of cocoa grown in Antioquia, Colombia.

**Fig. 2:**
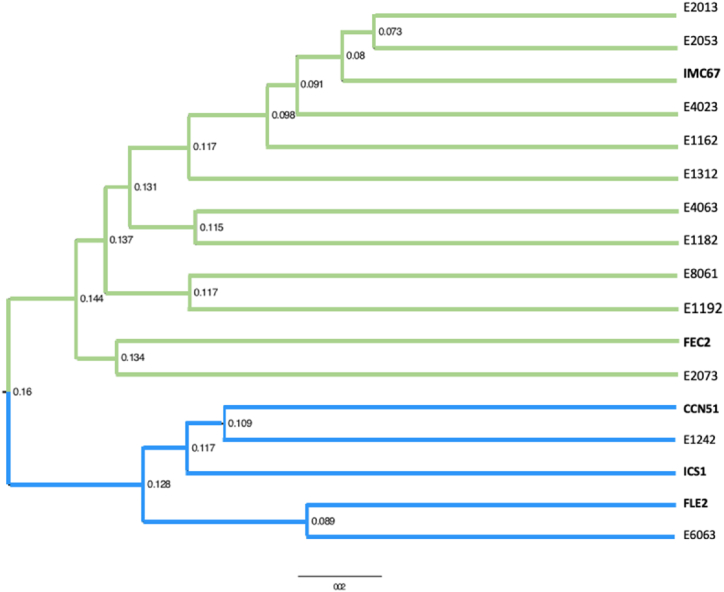
Dendrogram of genetic distances according to the Rogers standard (1972)(35) and the UPGMA algorithm.

The following SNPs were excluded from the set of 96 SNPs due to a high amount of missing data: TcSNP353, TcSNP1383, and TcSNP189. The final 93 markers were shown to be polymorphic, with an average PIC of 0.299. The obtained dendrogram (Fig. 2) had a maximum distance between samples of 0.18 using a range of distances from 0 to 1. Accordingly, the materials were considered to be closely related to each other. Some groupings were observed, although with little genetic distance between individuals. Genetic subgroups are highlighted in color, with the respective distances between nodes shown.

**Fig. 3:**
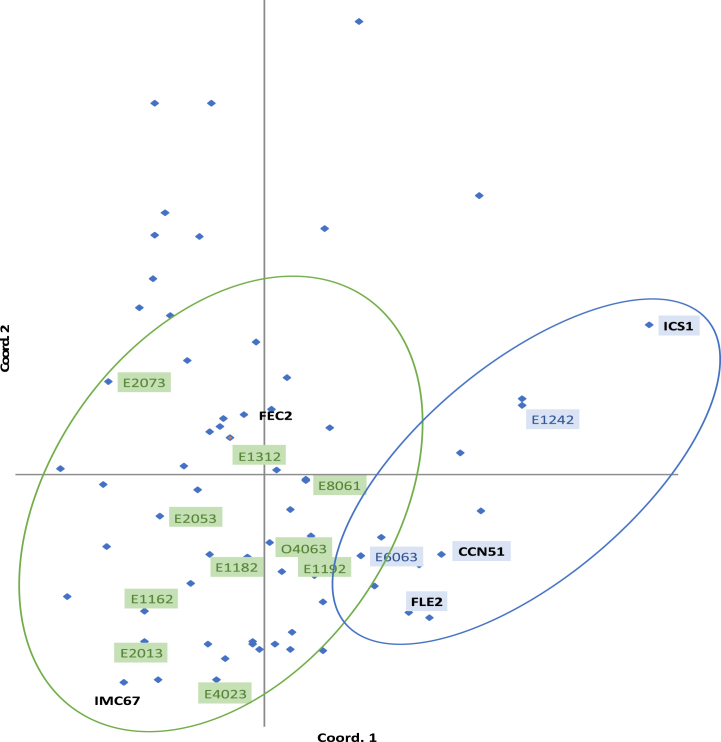
Principal coordinate.

Fig. 3 shows the principal coordinates analysis for the materials evaluated in the present study. The genetic groups are encircled by colors corresponding the groups identified in the distance dendrogram. Most materials promissories were found in a group containing the international reference clone IMC67 and the regional reference clone FEC2 (green). The reference clones CCN51, ICS1, and FLE2 and the promissories materials E6063 and E1242 were located in the other group (blue).

#### 3.1.2 Heterozygosity analysis of materials

**Fig. 4:**
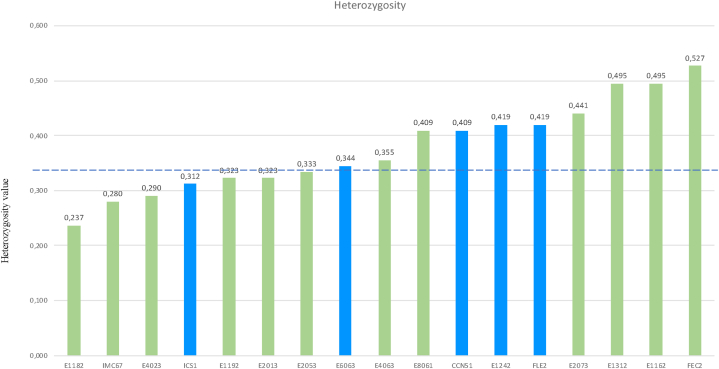
Heterozygosity analysis (H) for each material and reference cocoa.

Fig. 4 shows the heterozygosity of promissories materials and commercial references. The horizontal line corresponds to the average heterozygosity observed between the 68 samples evaluated in the present study (H = 0.338). Of the commercial clones, clone FEC2 (H = 0.517) and the clones belonging to the promissories E1162 and E1312 (H = 0.495 for both) had a particularly high heterozygosity. On the other hand, the international clones IMC67 and ICS1 had the lowest heterozygosity (H = 0.280 and H = 0.312, respectively). Among the promissories materials, clone E1182 had the lowest heterozygosity (H = 0.238).

Ancestral Criollo varieties (ssp. cacao) are known to be more susceptible to diseases and adverse environmental factors. Crossbreeding of Criollo varieties with other genetic varieties, particularly more resistant individuals of the Forastero variety, has allowed the genetic heritage of Criollo varieties to be preserved over time, giving rise to the so-called modern Criollo varieties. Motamayor et al. [4] reported low observed heterozygosity (H = 0.002) using RFLP markers among 92 cocoa samples classified as Cacao Criollo Ancestral collected in Venezuela, Mexico, Colombia, Nicaragua, and Belize, while the samples classified as Cacao Criollo Moderno found mostly in Venezuela, Mexico, and Trinidad had comparatively higher heterozygosity according to analyses using RFLP (H = 0.47) and microsatellite (H = 0.59) markers. In the same study, the highest heterozygosity when using microsatellite markers was observed among samples belonging to Cacao Forastero (H = 0.68). According to these results, the materials evaluated in the present study had similar heterozygosity to modern Criollo cocoas.

### 3.2 Biochemical and sensory characterization

Table 2 shows the chemical and sensory composition of reference clones and the 12 promissories materials from Antioquia.

**Table 2:** Chemical and sensorial scores of evaluated materials and reference clones.

Accession/collection	Ethereal extract, EE (%)	Total polyphenolic content (GAE, mg/100 g)	Total anthocyanin content (430/530 nm ratio)	ORAC (μmol TE/100 g)	Theobromine (mg/100 mg)	Caffeine (mg/100 mg)	Theobromine/caffeine ratio	Sensorial quality
CCN51	41.4	5218.4	1.0	16972.6	24.5	3.4	7.2	6
ICS1	42.7	5141.4	1.1	15465.2	25.1	4.9	5.1	5,5
IMC67	55.0	6793.0	2.1	34451.7	25.5	3.1	2.3	6,6
FLE2	53.2	5859.7	1.1	17363.3	22.4	6.5	3.4	7
FEC2	41.5	3635.3	1.0	10461.9	22.5	3.5	6.4	6.5
E8061	54.0	14286.9	1.5	36624.5	28.0	3.6	7.8	7
E1162	54.4	6835.9	1.4	32217.8	28.7	4.9	5.8	8
E1182	53.9	11047.8	0.9	44308.1	21.9	1.2	17.7	6,5
E1192	52.2	7466.7	1.6	35243.9	24.3	3.4	7.1	6
E1242	56.4	4276.0	1.46	20370.3	21.1	7.0	3.0	8
E1312	49.6	6621.9	1.0	25421.2	27.4	3.0	9.3	8,2
E2013	58.9	6138.3	1.7	24706.4	22.5	3.3	6.8	8
E2053	56.5	6504.6	1.7	24638.2	25.2	3.2	7.9	6,5
E2073	48.2	5863.5	1.4	24227.6	21.4	3.0	2.1	8,5
E4023	55.6	7202.8	1.6	28736.4	21.3	2.7	7.8	7,5
E4063	51.4	7536.2	1.5	30188.5	23.8	7.2	3.3	7,5
E6063	45.4	3701.3	1.7	27674.6	22.9	2.2	10.2	7

CCN51: Colección Castro Naranjal, ICS1: *Imperial Collage Selection*, IMC67: Iquitos Mezclado con Calabacillo. FLE2: Fedecacao Lebrija. FEC2: Federación El Carmen.

#### 3.2.1 Ethereal extract percentage

Ethereal extract percentage (%EE) is important in characterizing the quality of cocoa butter is this raw material has high economic value in the food, cosmetic, and pharmaceutical industries [40]. In the chocolate industry specifically, %EE is associated for the texture, plasticity, rheology, flavor diffusion, brightness, touch, and melting characteristics of derived products [41].

The %EE of cocoa is an indicator of outstanding quality, with a percentage greater than 50 % often used according to the results of previous studies [28,42]. This quality indicator was higher among the materials evaluated compared to the reference materials. For example, sample E6063 had a minimum %EE that was 4 % greater than FEC-2. Likewise, the maximum ethereal extract content of the E2013 sample was 58.9 %, which was 3.9 % greater than the reference material IMC 67. In addition, the E2013 material had a similar %EE to the universal clone TSH-565 which is used as a reference material due to a higher fat content of approximately 61 % [43].

#### 3.2.2 Total anthocyanin content

The anthocyanins 3-β-galactosyl-cyanidins and 3-α-l-arabinose-cyanidins are reportedly the main determinants of the color of raw cocoa beans [44]. A previous study by Niemenak et al. [45] demonstrated that anthocyanin content can be used to distinguish between cocoa clones by forming hierarchies according to anthocyanin concentration. Among the materials analyzed in the present study, total anthocyanin content was associated with cocoa variety. Accordingly, values below 1 (430/530 nm ratio) were classified as a distinctive indicator of possible Criollo cocoas, an aspect that contributes to the profiling of materials considered fine aroma cocoas. E1182 was classified as a possible Criollo cocoa or corresponding to an Albino cocoa [46] due to a low anthocyanin content; however, its sensory profile was not higher than 7 and it had a high theobromine/caffeine ratio. Criollo cocoa pods are traditionally associated with fine and aroma cocoa. Cocoa trees with these characteristics are typically found in Venezuela and Peru where white seed cocoa, which does not contain anthocyanins, has begun to be studied.

#### 3.2.3 Alkaloid content

The measurement of alkaloids, specifically theobromine (TB) and caffeine (CF), in cocoa is of great importance as the bitter taste of chocolate has been attributed to these components [25,28,47]. In addition, these compounds have been shown to have health benefits, particularly their properties as stimulants of the central nervous system. Further, TB and CF are considered mild diuretics and respiratory stimulants [48]. Alkaloids have also been used to classify cocoa varieties based on the theobromine/caffeine ratio [[49], [50], [51]]. Theobromine/caffeine ratios below 3, between 3 and 9, and between 9 and 11 correspond to Criollo cocoas, Trinitarios cocoas, and Forasteros cacaos; respectively.

According to the classification described by Davrieux et al. [49], theobromine/caffeine ratios could be used to classify three materials as Criollo cocoas (E2073, E1242, and E4063) and eight materials as Trinitarios (E1162, E2013, E1192, E8061, E4023, E2053, E1312, and E6063), which matched the four reference clones. Other authors [27,52] have highlighted the importance of determining the theobromine/caffeine ratio of cocoa material in greater detail. These studies used Forastero cocoa from Ghana and observed a high theobromine content (between 0.8 and 1.0 %) and a low caffeine content (between 0.1 and 0.2 %), unlike Venezuelan cocoa beans which have a low concentration of theobromine and a high caffeine content (between 0,3 and 0,4 %). This behavior influenced the Theobromine/caffeine ratio, in such a manner that ratios between 10 and 12 were reported for Forastero and between 1 and 4 for Venezuelan cocoa.

Due to the complex diversity of cocoa clones worldwide, studies such as the one conducted by Motamayor et al. [53], indicate that cocoa clones can be classified into 10 genetic groups (Marañon, Curaray, Criollo, Iquitos, Nanay, Contamana, Amelonado, Purús, Nacional, and Guayana) rather than the three traditional groups (Criollos, Trinitarios, and Forasteros). A recent study proposed a different classification method based on methylxanthines concentration where low theobromine content and high caffeine content correspond to Criollo and the opposite corresponded to Forasteros, while Trinitario genotypes have intermediate theobromine and caffeine contents [54,55]. The E2073, E1242, and E4063 materials are classified as possible Criollo genotypes when the above classification method is used, with a lower theobromine/caffeine ratio associated. In contrast, the material E1182 whose TB/CF ratio is the highest found among the materials studied is classified as Forastero cocoa.

The optimal parameters for alkaloid extraction from raw cocoa beans were an extraction duration of 40 min and ultrasound frequency of 40 Hz, which resulted in a recovery percentages of 103 % for theobromine and 106 % for caffeine.

Use of the optimized extraction conditions for alkaloid extraction from SRM 2384 - Baking Chocolate (NIST, USA) as a reference material validated the effectiveness of this method, with an extracted theobromine content of 12555 mg/kg, which was within the expected range of 10500 and 12700 mg/kg. Similarly, the concentration of caffeine extracted from the reference material was 1043 mg, a value within the expected range of 1010–1110 mg/kg.

It should be noted that the measured methylxanthine contents in the present study (Table 1) are above the maximum values reported for cocoa from Peru (TB, 73.59 ± 2.03 mg/g; CF, 4.10 ± 0.3 mg/g) [56], Mexico (TB, 21.97 ± 0.03 mg/g) [57], Colombia (TB, 7.12 ± 0.15 mg/g; CF: 2.31 ± 0.03 mg/g) (Borja et al., 2022), and Africa (TB, 24.74 ± 2.20 mg/g) [58].

These differences in the alkaloid content of cocoa beans from different countries confirm hypotheses regarding the influence of edaphological conditions, climate, and agronomic management [59]. The different ecotypes resulting from crossing of Forastero or Amazonico with Trinitario clones have resulted in greater genetic variability with characteristic profiles according to region [21]. The results of the present study conducted in the same state with varied agro-climatic conditions are in-keeping with these hypotheses.

#### 3.2.4 Total polyphenolic content and antioxidant capacity

The correlation analysis between the total polyphenolic content and antioxidant capacity evaluated according to the ORAC method resulted in an r value of 0.7595, which indicates a strong positive correlation. Compared to studies from other regions, this correlation is lower than observed for other fermented cocoa matrices [58]. This difference in the association between total polyphenolic content and antioxidant capacity may be due to the availability of phenolic compounds or interactions between phenolic compounds and proteins. Further, macronutrient content may be affected by the use of fertilizers, which may vary significantly given the varying edaphoclimatic conditions between the regions from which the materials were obtained. In addition, the determination of antioxidant capacity using the ORAC method relies on the trapping capacity of hydroxyl radicals, which has also been attributed to theobromine [60]. Accordingly, theobromine content is not included in the total content of polyphenols as it is a methylxanthine [61].

Due to the above, it the measurement of phenolic compounds is not the best tool for the differentiation of cocoa clones, with the results of previous studies [45] supporting this conclusion. However, the measurement of phenolic compounds is important as they influence the quality of cocoa and are strongly related to the astringent flavor [62] and functional properties [63] of cocoa clones, indicating the importance of determining phenolic content in profiling cocoa materials. The materials with the highest antioxidant capacity in the present study were E1182, E8061, and E1192, which had characteristics in-keeping with the profile of Forastero cocoa. These materials also had a high total polyphenol content between 7466.7 and 14286.9 mg GAE/100 g, which can explain astringent flavor characteristics of these bioactive compounds. These materials appear to have a set of genotypes with similar functional properties to the hybrids classified as Forasteros by Borja et al. [54], which had maximum total polyphenol contents between 7796 ± 39.4 and 10677 ± 521 mg GAE/100 g. The results of the present study and those of the previous study by Borja et al. that used CCN51 as a reference Forastero clone highlight the potential functional profiles of E1182, E8061, and E1192.

### 3.3 Multivariate statistical analysis

**Fig. 5:**
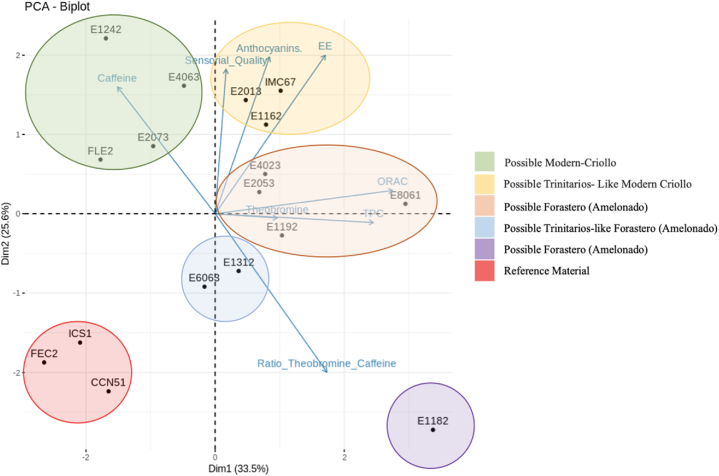
Biplot of the PCA obtained from the chemical and sensory profile of cocoa materials from Antioquia, Colombia presented in a factorial plane composed of two main components that explain 59.1 % of the total variance.

Global analysis of the chemical and sensory tests for classifying cocoa materials from Antioquia was performed using principal component analysis, which resulted in the formation of a series of subgroups according to certain salient variables. The contributions of parameters conducive to variability in the first two principal components are presented in Fig. 5.

The PCA approach allows the identification of variables significantly associated with the 12 cocoa hybrids analyzed. The distribution of the variables throughout the first and second principal components is most important when explaining variability in the data set and when grouping materials according to the chemical or sensory parameters that best explain identity characteristics.

The grouping of the selected samples can be used to classify cocoa from Antioquia, as Criollo, Trinitarios, or Forasteros according to similarity or compliance with the standards proposed by previous studies, as described above. The first group (green) included the E1242, E2073, and E4063 samples which were characterized by a high caffeine concentration associated with Criollo cocoa [47,64,65]. In addition, these samples had high sensory quality values and low theobromine content, which contributed to a low theobromine/caffeine ratio, in addition to low total polyphenolic content. Sample E1242 had a high ethereal extract content, which indicates E1242 is a very promising material from the perspective of fine aroma and may be of interest to industrial applications. Finally, this group included one of the reference cocoas registered in Colombia, FLE-2 (Fedecacao Lebrija, Santander), which is characterized by its capacity for agronomic adaptation with high productive yields. FLE-2 is particularly notable for having important sensory attributes due to its mild fruity aroma [66].

The second group (yellow) included materials E2013 and E1162 due to similar anthocyanin content, ethereal extract percentage, and sensory quality. These materials had characteristics associated with Trinitario, including high sensory quality and a theobromine/caffeine ratio <7, the value within the characteristic range of Trinitario cocoa (3–9). Nevertheless, the anthocyanin content of E2013 was high (>1) and E1162 had the highest theobromine content and was therefore not classified as Criollo. However, these values highlight the sensory attributes of these materials may have utility in producing derivatives with outstanding aromas and flavors. This group also included the reference cocoa IMC67, which is used as a clone for patterning due to its resistance to diseases [67].

The group shown in orange had high total polyphenolic content, ORAC, and theobromine content, properties specific to Forasteros cocoa. Accordingly, materials E4023, E2053, E8061, and E1192 were proposed as belonging to this variety of cocoa. E2053 and E4023 had particularly high values for ethereal extract percentage, which indicate these materials may be of interest to industrial applications. E1312 and E6063 were explained by the theobromine and caffeine ratio and are shown in blue in Fig. 4. This group, opposite to the green group, had a high theobromine content and low caffeine content but low total polyphenolic and anthocyanin content, thereby placing these materials in the group of Trinitarios-like Forasteros, particularly as they had a sensory quality greater than 7. On the other hand, E1182 was placed into the PC1 and PC2 quadrants and classified into a different group due to a theobromine/caffeine ratio within the range of Forasteros cocoas. The reference materials CCN-51, ICS-2, and FEC-2 were grouped in the lower quadrant of PC2 as they had significantly different chemical and sensory profiles to other materials.

**Fig. 6:**
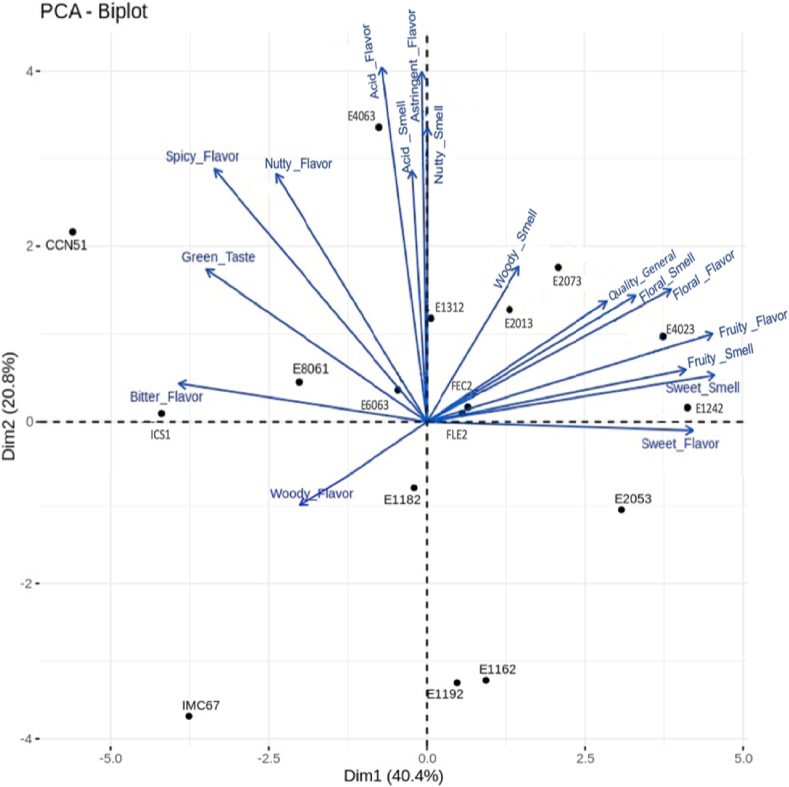
Biplot of the sensory profiles obtained from PCA for cocoa genotypes from Antioquia, Colombia.

Finally, a PCA was developed using sensory odor and flavor descriptors of cocoa materials to establish the association of these descriptors with the groupings shown in Fig. 6. Component 1 explained 40.4 % and component 2 explained 20.8 % of the total variability. Materials E1242, E2073, and E4063 that make up the first group (green) are related to sensory descriptors of fruit-type odor and flavor associated with citrus, red fruits, and banana and floral notes (rose and sweet). In the second group (yellow), the E2013 material was classified as Trinitarian and possibly modern Criollo due to its high sensory quality that was associated with fruity notes (banana, apple, and citrus) and a high flowery odor (rose). Materials classified as foreigners such as E4023, E2053, E8061, and E1192 were associated with sensory descriptors including acid, bitter, astringent, and pungent that negatively impacted the aromatic and flavor qualities of these materials ([47,64,65]).

### 3.4 Analysis of factors obtained from the principal components analysis

To determine the intentionality of the information obtained for the chemical and sensory profiles of cocoa materials with the potential to be registered as official planting materials or used as quality controls material in agronomic studies, the components obtained from PCA were used for principal factors analysis (PFA-PCA).

The analysis led to the creation of three groups that could be used as a tool for making decisions regarding the characterization and distinction of the unidentified cocoa materials from Antioquia, Colombia according to the characteristics previously obtained from PCA of chemical and sensory properties.

Table 3 present the three factors with respective loadings and the unobservable denomination that was reached. The three factors together explained 100 % of the variation of the whole sample analyzed, with a weights of 0.20, 0.63, and 0.63 for Factor 1, Factor 2, and Factor 3, respectively. Factor 1, termed “cocoa variety distinction” included variables that distinguish Criollos and Forasteros cocoas including the high caffeine content characteristic of Criollo cocoas and the high theobromine/caffeine ratio associated with Forasteros cocoas [54]. This grouping makes it possible to identify the chemical properties that defined this type classification at a decision-making level according to the intended use.

**Table 3:** Analysis of the predominant characteristics of unidentified cocoa materials from Antioquia, Colombia.

Factor (Weighted average-loadings)	Proportion of explained variance	Properties and loadings	Genotype	Proposed classification
Cocoa variety distinction (0.20)	0.28	Theobromine/caffeine ratio [1]	E1312, E6063, E1182*	Forastero Amelonado
		Caffeine (−0.6)	E1242, E4063, E2073, FLE2	Modern Criollo
Productive performance and outstanding sensory profile (0.63)	0.36	Ethereal extract [1]	E2013, E1162, IMC67	Trinitario
		Anthocyanins (0.5)		
		Sensorial Score (0.4)		
Functional properties (0.63)	0.36	TPC (0.8)	E1192, E2053, E8061, E4023	Forastero Amelonado
		Orac (0.6)		
		Theobromine (0.5)		

Factor 2 was termed “productive performance and sensory quality profile.” In the first instance, the product performance was related to a high ether extract percentage, which has been recognized for its importance in the development of cocoa derivatives with high plasticity and commercial value. The second aspect associated with Factor 2 was sensory quality as the materials that contributed to this factor, E2013 and E1162, were distinguished by a sensory quality score of 8, which is outstanding and coincides with the scores of Trinitarios or Criollo cocoa.

Factor 3, termed “functional properties,” was defined according to properties associated with antioxidant capacity and total polyphenolic and theobromine contents, which can be used for the development of derivatives with a functional profile that fulfil the demand for new healthy foods. However, high TPC has a significant contribution to astringent taste, which increases the challenge of achieving a balance in taste profile when used these materials [68].

The results of the present study demonstrate relationships between the molecular characteristics of cocoa (genetic group and SNP heterozygosity), antioxidant capacity (TPC and ORAC), alkaloid content predominantly related to theobromine (TB) and caffeine (CF), anthocyanin concentration, and ethereal extract percentage of cocoa beans, which confer important sensory and agronomic characteristics to the materials obtained from farms in Antioquia, Colombia. It is expected that it can be a comprehensive methodology adopted by cocoa producers and processors that allows them to carry out the traceability and propagation of possible materials with true commercial interest, which also has functional properties currently in demand by the food, pharmaceutical and cosmetic industries.

Although establishing quality criteria through the evaluation of genetic, chemical and sensory properties in the cocoa bean is key to developing greater competitiveness and positioning in differentiated markets, subsequent work is required where instrumental techniques such as olfactometry are used, which allows us to associate the odor activity value (OAV) in the cocoa bean and the development of aromatic compounds associated with genetic groups.

In summary, the evaluation of molecular, biochemical, and sensory characteristics of unlabeled cocoa materials may facilitate the selection of valuable materials for cocoa plant breeding programs anywhere in the world.

## Data availability statement

Data associated with the study has not been deposited into a publicly available repository and data will be made available on request.

## CRediT authorship contribution statement

**Andrea Zapata-Alvarez:** Writing – review & editing, Writing – original draft, Methodology, Investigation, Formal analysis, Conceptualization. **Carolina Bedoya-Vergara:** Writing – review & editing, Writing – original draft, Methodology, Investigation, Conceptualization. **Luis D. Porras-Barrientos:** Writing – original draft, Methodology, Investigation. **Jessica M. Rojas-Mora:** Writing – original draft, Formal analysis, Data curation. **Héctor A. Rodríguez-Cabal:** Writing – review & editing, Writing – original draft, Validation, Supervision, Project administration, Methodology, Investigation, Formal analysis, Conceptualization. **Maritza A. Gil-Garzon:** Writing – review & editing, Project administration, Methodology, Investigation, Formal analysis, Conceptualization. **Olga L. Martinez-Alvarez:** Writing – original draft, Supervision, Project administration, Methodology, Investigation. **Carlos M. Ocampo-Arango:** Methodology, Investigation, Conceptualization. **Maurem P. Ardila-Castañeda:** Methodology, Investigation, Formal analysis. **Zulma I. Monsalve-F:** Methodology, Investigation, Formal analysis.

## Declaration of competing interest

The authors declare that they have no known competing financial interests or personal relationships that could have appeared to influence the work reported in this paper.
